# Emergent carotid artery stenting with tirofiban in a patient with traumatic intracranial hemorrhage and carotid artery dissection: a case report

**DOI:** 10.3389/fmed.2025.1626194

**Published:** 2025-08-13

**Authors:** Shu Yang, Jian-Hong Wang, Bin Huang, Fu-Qiang Guo, Bing-Hu Li

**Affiliations:** Department of Neurology, Sichuan Academy of Medical Sciences and Sichuan Provincial People's Hospital, Chengdu, China

**Keywords:** acute cerebral infarction, carotid artery dissection, case report, endovascular therapy, emergent carotid artery stenting, traumatic intracranial hemorrhage

## Abstract

Endovascular therapy (EVT) is an effective treatment for large vessel occlusion, including carotid artery dissection (CAD). However, when large vessel occlusion (LVO) occurs in acute ischemic stroke (AIS) patients with concomitant intracranial hemorrhage (ICH), it can be difficult for clinicians to make a treatment decision. We report the case of a patient in their 60s who was admitted due to sudden right limb weakness. Emergency computed tomography (CT)/CTA showed a hematoma in the right parietal lobe and a possible dissection of the left internal carotid artery. The patient underwent emergent carotid artery stenting (eCAS) followed by postoperative tirofiban administration. At six-month follow-up, there was no evidence of new bleeding, clinical function had recovered, and no re-stenosis was observed. For patients with AIS due to CAD complicated by ICH, eCAS combined with tirofiban may offer clinical benefits. However, further research is still needed to confirm our findings.

## Introduction

Endovascular therapy (EVT) is an effective treatment for patients with acute ischemic stroke (AIS) due to large vessel occlusion (LVO) (1). In clinical practice, patients with acute ischemic stroke (AIS) who develop concurrent intracranial hemorrhage (ICH) may be excluded from EVT. However, a meta-analysis indicated that EVT is feasible in AIS patients with concurrent intracranial hemorrhage (ICH), although it is associated with poor functional outcomes and high mortality rates (2). It still needs to be determined which patients are suitable for EVT. Regarding the etiology of LVO, carotid artery dissection (CAD) is one of the common causes (3). The primary treatments for CAD are antithrombotic therapies using either anticoagulants or antiplatelet drugs (4). EVT is only required in cases of symptomatic cervical artery stenosis or occlusion. A recent study revealed that EVT may improve functional outcomes in patients with LVO due to CAD and an admission National Institutes of Health Stroke Scale (NIHSS) score ≥6 but not in those with an NIHSS score <6 (5). However, it is still unclear whether EVT or the best medical treatment is more suitable for patients with AIS and CAD. Clinically, when patients have AIS and concurrent CAD with ICH, it is difficult for clinicians to make decisions. Here, we present the case of a patient in their 60s with traumatic ICH who developed AIS due to CAD.

## Case presentation

A patient in their 60s was admitted due to the sudden onset of right-sided limb weakness and speech difficulties lasting for 5 h. Physical examination revealed drowsiness, left eye deviation, aphasia, muscle strength of grade 2 in the right limbs and left lower limb, and a left upper limb fracture. The NIHSS score was 22.

The medical history was unremarkable. Furthermore, five days before, the patient was involved in a car accident, resulting in a fracture of the left upper limb (a distal linear fracture of the left radial bone and a fracture of the left ulnar styloid process, both without significant displacement) and an intracranial hemorrhage in the right parietal lobe. A non-contrast head computed tomography (CT) scan revealed a hematoma measuring 23 mm × 23 mm. The patient subsequently developed left-sided limb weakness, with muscle strength of grade 2. The patient received external fixation for the left arm fracture and no specific treatment for the cerebral hemorrhage, aside from blood pressure control, with blood pressure maintained below 140 mmHg. The patient denied a history of hypertension, diabetes, or smoking. There was no family history of genetic disorders.

**Figure 1:**
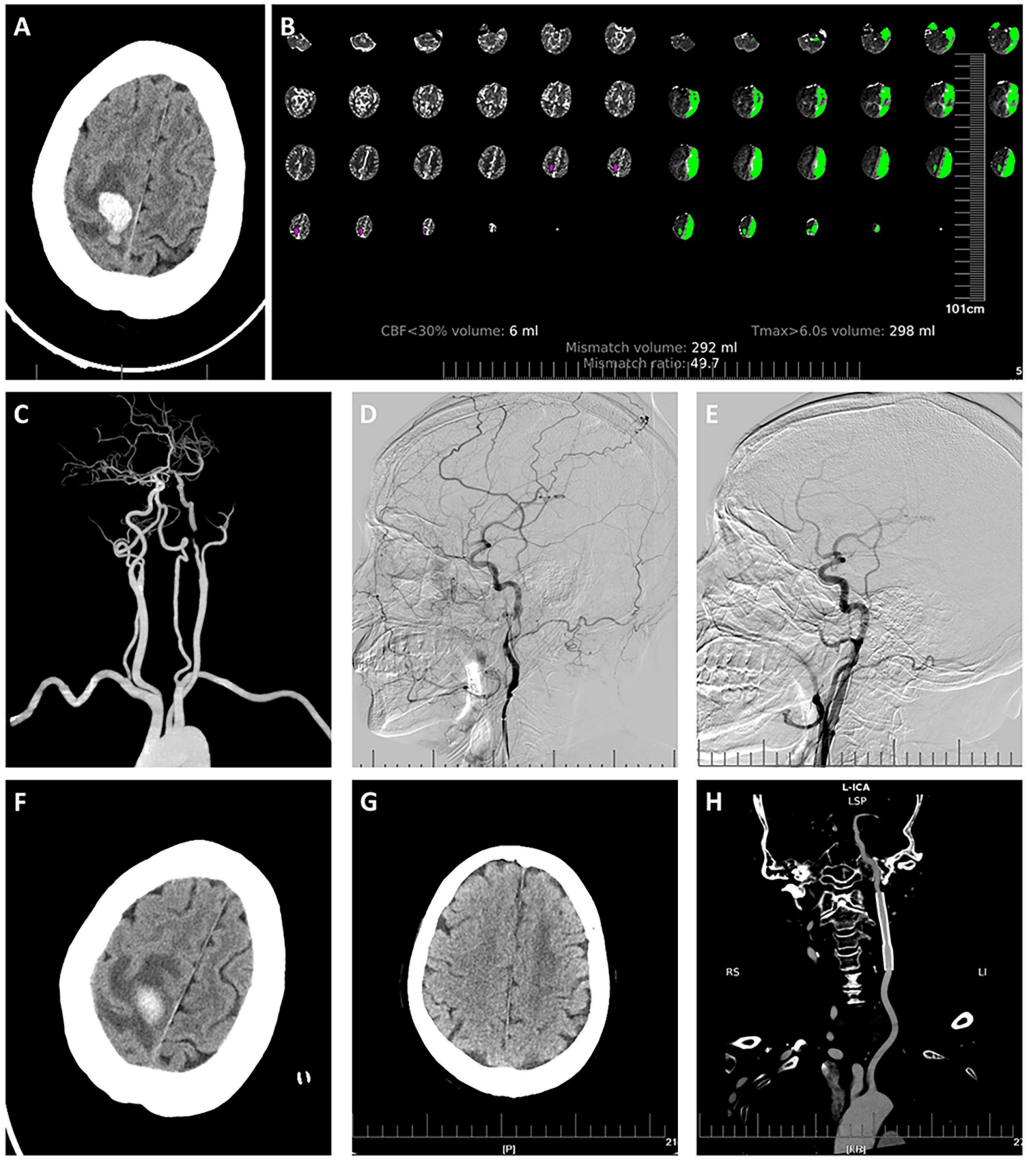
Brain images of the patient. **(A)** Emergency head CT showed a subacute right parietal lobe hematoma measuring 23 mm*23 mm*12 mm. **(B,C)** Emergency head and neck CTA and CTP. CTA indicated severe stenosis at the C1 segment of the left internal carotid artery. CTP showed a low perfusion area of 298 mL and a mismatch of 49.7. **(D)** Emergency angiography on admission showed an intimal flap in the middle section of the C1 segment of the left internal carotid artery. **(E)** Immediate angiography post-stenting. **(F)** Head CT 9 days after stenting. G: Head CT 6 months after stenting showed absorption of the hematoma. **(H)** Head and neck CTA 6 months after stenting showed no signs of narrowing in the stent.

Emergency non-contrast computed tomography (CT) showed a subacute hematoma in the right parietal lobe (23 mm*23 mm*12 mm) (Figure 1A). Head CT perfusion (CTP) showed a low perfusion area of 298 mL and a mismatch ratio of 49.7 (Figure 1B). Head and neck CTA indicated severe stenosis at the C1 segment of the left internal carotid artery (Figure 1C). Further digital subtraction angiography (DSA) showed an intimal flap on the middle section of the C1 segment of the left internal carotid artery. Considering the patient’s history of trauma and the findings from head and neck CTA, there was a high possibility of carotid artery dissection (CAD) caused by trauma (Figure 1D).

Given the medical history, imaging findings, and clinical manifestations, a multidisciplinary team—including neurology, neurosurgery, and critical care medicine—suggested that the patient’s new neurological symptoms were caused by AIS due to left internal CAD. The patient was at high risk for cerebral ischemia, cerebral edema, and even cerebral herniation. Internal carotid artery recanalization is currently considered the best treatment option for this patient. The patient had a 5-day history of traumatic cerebral hemorrhage, with no evidence of hematoma enlargement on CT re-examination, and a low risk of bleeding assessed by the HAS-BLED score (1 point). The final treatment plan included emergent carotid artery stenting (eCAS) followed by antiplatelet drugs (6). Given the advantages of a shorter plasma half-life and rapid recovery of platelet function after discontinuation, tirofiban was selected as the antiplatelet drug.

After obtaining informed consent from the patient’s family members, eCAS was performed using a Wallstent 9*50 mm carotid stent (Figure 1E) and tirofiban was administered. The dose of tirofiban was calculated at 0.1 μg/Kg/min. For a patient weighing 50 kg, the dose of tirofiban should be 6 mL/H. However, due to concerns about hematoma enlargement, a relatively lower dose of 4 mL/H was administered continuously.

On the 9^th^ day post-stenting, the patient showed improvement in right limb muscle strength to grade 4 while receiving continuous tirofiban infusion at 4 mL/H. A head CT scan was performed, which showed absorption of the hematoma (Figure 1F). Given the absorption of the hematoma and the absence of new bleeding, as well as the better prevention of stent thrombosis, the antithrombotic treatment was planned to transition to oral dual antiplatelet therapy. Considering clopidogrel resistance, tirofiban was replaced with dual antiplatelet therapy with aspirin (100 mg/day) plus ticagrelor (90 mg/Bid) (7). The use of tirofiban was discontinued 4 h after the oral administration of aspirin and ticagrelor. Aspirin plus ticagrelor was planned to be switched to aspirin monotherapy after 6 months (8).

On the 45^th^ day post-stenting, the patient showed progressive improvement in neurological symptoms but still had mild dysarthria at the time of discharge. The patient was fully conscious, with slightly slurred speech, right limb muscle strength of grade 5, left upper limb weakness, left lower limb muscle strength of grade 2, an NIHSS score of 5, and a modified Rankin Scale (mRS) score of 3.

At six-month follow-up, right limb muscle strength had fully recovered to grade 5. Head CT/CTA (Figures 1G,H) showed that the hematoma had mostly resolved, with no signs of narrowing in the stent.

## Discussion

Although EVT is an effective treatment for patients with AIS due to LVO, there are no recommendations for AIS patients with concurrent ICH. A meta-analysis (2) including six studies and 49 patients demonstrated that the overall incidence rate of successful revascularization was 85.3% and functional independence was achieved in 20% of patients. This meta-analysis suggests that EVT is feasible in AIS patients with concurrent ICH; however, it is associated with poor functional outcomes and high mortality rates. In the original studies included in this meta-analysis, no LVO was due to CAD and no emergent carotid artery stenting (eCAS) was performed. In our case of traumatic ICH complicated by CAD, the decision between performing EVT—with its risk of hemorrhage progression—and opting for conservative treatment—with its risk of poor clinical outcomes—posed a significant dilemma.

CAD is a common cause of stroke, accounting for up to 25% of cases in adults under 50 years of age (7). eCAS for CAD remains controversial. Cervical artery dissection is a relatively uncommon complication following trauma. For patients without hypoperfusion, eCAS may be unsuitable (7). However, previous studies have demonstrated that eCAS may improve distal perfusion in patients with neurological deficits due to hypoperfusion (9). Therefore, for this case, eCAS was considered reasonable. However, another issue that needs to be considered regarding this case is the periprocedural antithrombotic regimen.

For eCAS, appropriate antithrombotic treatment is indispensable, as early in-stent thrombosis may occur in 5–20% (10), but the optimal antithrombotic regimen still needs to be determined. A retrospective single-center study investigating the efficacy and safety of tirofiban compared to aspirin in patients with AIS undergoing eCAS showed that periprocedural antithrombotic therapy with tirofiban was associated with a lower risk of in-stent thrombosis and sICH within 24 h after eCAS compared to aspirin (11). Another meta-analysis including 34 studies involving 1,658 patients suggested that good functional outcomes are comparable across different antithrombotic treatment regimens, with trends favoring glycoprotein IIb/IIIa inhibitors over dual or single antiplatelet therapy in terms of good functional outcomes (12). This further supports the efficacy and safety of tirofiban. Therefore, the patient in this case was administered tirofiban immediately after eCAS. Follow-up brain CT showed no new bleeding and no enlargement of the previous hematoma.

A limitation of this report is that it is based on a single case, so the findings cannot be generalized. In particular, the safety of tirofiban in patients with acute ICH has not been established, and the risk of hemorrhagic complications is considered unacceptably high in this context.

In conclusion, for patients with AIS due to CAD complicated by ICH, eCAS with tirofiban may offer clinical benefits. This case provides a reference for clinicians to make decisions when faced with similar cases. Although previous studies suggest that EVT is feasible in AIS patients with concurrent ICH, it is associated with poor functional outcomes and high mortality rates. Therefore, our results require further research for confirmation.

## Data Availability

The raw data supporting the conclusions of this article will be made available by the authors, without undue reservation.
